# A nomogram prediction model of pseudomyxoma peritonei established based on new prognostic factors of HE stained pathological images analysis

**DOI:** 10.1002/cam4.7101

**Published:** 2024-03-20

**Authors:** Ru Ma, Yan‐Dong Su, Feng‐Cai Yan, Yu‐Lin Lin, Ying Gao, Yan Li

**Affiliations:** ^1^ Department of Peritoneal Cancer Surgery, Beijing Shijitan Hospital Capital Medical University Beijing China; ^2^ Department of Pathology, Beijing Shijitan Hospital Capital Medical University Beijing China; ^3^ Department of Surgical Oncology Beijing Tsinghua Changgung Hospital Affiliated to Tsinghua University Beijing China

**Keywords:** HE pathological images, nomogram, pathological features, prognosis, pseudomyxoma peritonei

## Abstract

**Background:**

Pseudomyxoma peritonei (PMP) is a rare clinical malignant syndrome, and its rarity causes a lack of pathology research. This study aims to quantitatively analyze HE‐stained pathological images (PIs), and develop a new predictive model integrating digital pathological parameters with clinical information.

**Methods:**

Ninety‐two PMP patients with complete clinic‐pathological information, were included. QuPath was used for PIs quantitative feature analysis at tissue‐, cell‐, and nucleus‐level. The correlations between overall survival (OS) and general clinicopathological characteristics, and PIs features were analyzed. A nomogram was established based on independent prognostic factors and evaluated.

**Results:**

Among the 92 PMP patients, there were 34 (37.0%) females and 58 (63.0%) males, with a median age of 57 (range: 31–76). A total of 449 HE stained images were obtained for QuPath analysis, which extracted 40 pathological parameters at three levels. Kaplan–Meier survival analysis revealed eight clinicopathological characteristics and 20 PIs features significantly associated with OS (*p* < 0.05). Partial least squares regression was used to screen the multicollinearity features and synthesize four new features. Multivariate survival analysis identified the following five independent prognostic factors: preoperative CA199, completeness of cytoreduction, histopathological type, component one at tissue‐level, and tumor nuclei circularity variance. A nomogram was established with internal validation C‐index 0.795 and calibration plots indicating improved prediction performance.

**Conclusions:**

The quantitative analysis of HE‐stained PIs could extract the new prognostic information on PMP. A nomogram established by five independent prognosticators is the first model integrating digital pathological information with clinical data for improved clinical outcome prediction.

## Abstract 2

Pseudomyxoma peritonei (PMP) is a rare clinical malignant syndrome, and its rarity causes the insufficiency of pathology research. This study established a prognosis nomogram prediction model based on new pathological characteristics of PMP through quantitative analysis of pathological images.

## 1 INTRODUCTION

Pseudomyxoma peritonei (PMP) is a malignant clinical syndrome characterized by the accumulation and redistribution of mucus produced by mucinous tumor cells (TCs) in the peritoneal cavity, with its typical clinical manifestations including mucinous ascites, peritoneal implantation, omental cake, and ovarian involvement in women.^1^ The overall incidence of PMP is 2–4/million, the prevalence is 25.1/million, the male to female ratio is 1:1.2–3.4, and the median age is 43–63 years old.^2^ Currently, cytoreductive surgery (CRS) plus hyperthermic intraperitoneal chemotherapy (HIPEC) is the standard treatment to significantly improve the survival.^3^

As a rare clinical tumor syndrome, the pathological classification and grading of PMP is a routine method to predict the biological characteristics and clinical outcomes of PMP.^4^, ^5^ At present, the recognized histopathological typing is mainly based on the qualitative assessment of TCs number, tumor nests (TNs) morphology, TCs atypia, mitotic, and tumor invasion mode.^6^ However, it has long been observed that some low‐grade mucinous carcinoma peritonei (LMCP) patients without any known adverse prognostic factors had poorer prognosis than some high‐grade mucinous carcinoma peritonei (HMCP) patients with poor prognostic features.^7^ Several factors could account for such complexity. First, such works is prone to observer differences among PMP pathologists. Second, due to the tumor heterogeneity, some patients have different histopathological types in different or even the same tumor sites, but with only one result in the final pathological diagnosis. Finally, the tumor microenvironment can mediate the occurrence and progression of PMP and cause morphological changes at the tissue‐ or cell‐level, which are visible in pathological images (PIs).^8^ Therefore, it is necessary to explore new pathological prognostic factors of PMP based on the analysis of in‐situ features in PIs.

The development of digital pathology has greatly promoted the application of image analysis research in pathology, and tumor pathology has gradually developed from manual qualitative diagnosis to machine learning‐associated diagnosis.^9^ The PIs analysis techniques developed on this basis can help overcome the inconsistency of subjective interpretation and help to explore new pathological prognostic features. Nowadays in the field of solid tumors, a variety of methods for PIs analysis, computer‐aided diagnosis and prognosis have been proposed, providing a new direction for the development of PMP pathological research.^10^, ^11^ This study aims to extract rich quantitative morphological features through digital PIs analysis, and integrate various clinical data to establish prognostic model to explore new pathological prognostic features.

## 2 MATERIALS AND METHODS

### 2.1 Patient cohort

A cohort of 92 PMP patients, who had no prior surgical history and received CRS + HIPEC at our center from December 2015 to December 2021, were included. Tissue slides, formalin‐fixed paraffin‐embedded tissue blocks, clinicopathological data, and follow‐up information were available. The other inclusion and exclusion were consistent with CRS + HIPEC criteria.^12^ All the surgical specimens and HE stained slides were reread by two senior pathologists (Yan FC, Gao Y) according to the Peritoneal Surface Oncology Group International (PSOGI) histopathological diagnostic criteria of PMP, and PMP was divided into three types: LMCP, HMCP, HMCP with signet ring cells (HMCP‐S).^6^ For the sake of model establishment, we have excluded the acellular mucin type. Overall survival (OS) was used as the primary endpoint and defined as the time from clinical diagnosis.

### 2.2 Tissue slides construction and scanning

All the conventional surgical specimens of PMP patients were subjected to thorough histopathological study, with routine HE staining (Dako Hematoxylin, Dako Eosin and Dako Bluing Buffer, catalog number CS701; Dako CoverStainer, Agilent Technologies Inc., USA). PMP specimens were collected from different anatomical regions, and five HE stained slides with most prominent tumor proliferation and aggressive growth were selected from each patient by a senior pathologist (Yan FC).

Tissue slides were subsequently scanned at 200 × magnification (0.246 μm/pixel) using a whole slide scanner (KF‐PRO‐400 scanner, Jiangfeng, China). Each slide was scanned into a whole‐slide images (WSI) with the image type of “.kfb files”.

### 2.3 Image preprocessing and ROI selection

First, the “.kfb files” were converted to “.svs files” using a format converter (Jiangfeng, China). And then scanned WSIs were imported into QuPath (v.0.3.2, University of Edinburgh, UK), an open‐source digital image analysis software. The color deconvolution algorithm in QuPath was used for stain separation to give a normalized representation of hematoxylin and eosin colors in the image, so that the subsequent image research could not be affected by different staining intensities of images as much as possible. Commands such as “Add smoothed features” and “Add intensity features” were performed to improve classification accuracy of detection of target areas.

The tumor areas with typical histopathological features, namely regions of interests (ROIs), were randomly selected for subsequently study by two investigators (Ma R, Yan FC), and then ROIs 500 × 500 μm (2029 × 2029 pixels) in size were generated within QuPath. ROIs included tumor and stroma areas, but not necrotic areas or improper staining artifacts. To ensure adequate representation of each patient and minimize selection bias, the goal of selecting a minimum of 25 patches per patient (five images per patient and five patches per image) was set.

### 2.4 Image features extraction

QuPath was carried out to segment tissue, and cell detection process, including the creation and manipulation of modules such as interactive mapping tools (annotating TNs and stroma) or automatic segmentation commands (such as detection TCs or nuclei).^13^

Manual annotation tools such as *Brush* and *Wand* were used to mark the TNs and stroma within the ROIs, and Command “Cell Detection” was used to quickly detect nuclei and cells. The detection parameters were unified as follows: background radius of nucleus was 8 μm; sigma was 1.5 μm; minimum and maximum area were 10 and 400 μm^2^; threshold of intensity was 0.1; maximum background intensity was 2; and other parameters were the default values. Then, any non‐cellular, folded, blurry or defective morphology were manually removed. The detected cells were appropriately defined as four components (including TCs, immune cells, blood cells, and other stromal cells) by module “Object classification”, and annotated accordingly by two investigators (Ma R, Yan FC) using annotation tools.

Then, the features of TNs, stroma, TCs, all stromal cells and nuclei were extracted from the tissue‐, cell‐ and nucleus‐level, respectively. The color features were greatly affected by the PIs themselves, so they were not included in this study. Since five different WSIs were collected for each patient, the average of the features in the five images was calculated as the final feature value.

### 2.5 Follow‐up

Follow‐up records included survival status and OS. The last unified follow‐up date was May 31, 2023, and the follow‐up rate was 100%.

**FIGURE 1:**
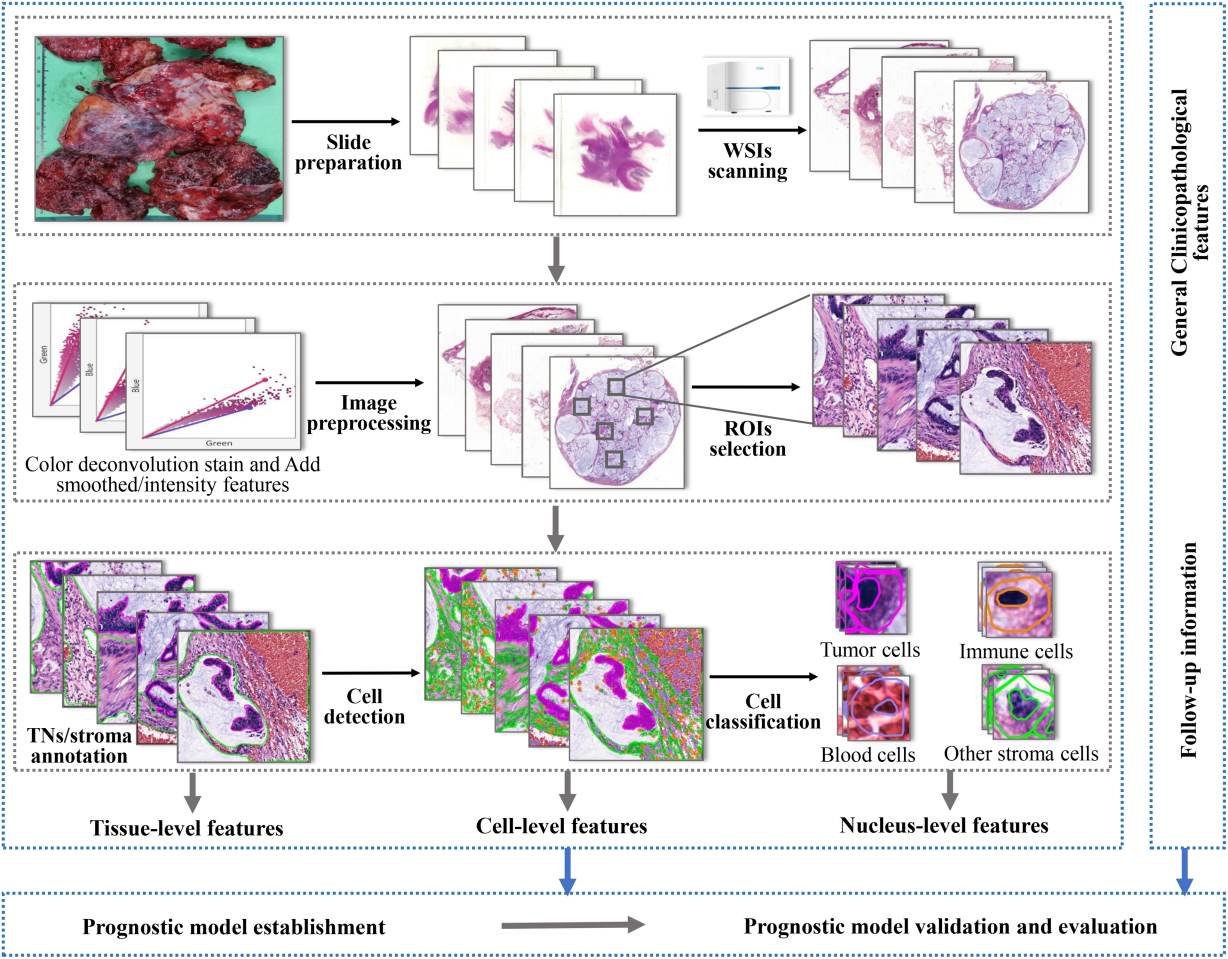
The workflow of pathological images (PIs)‐based prognostic model for PMP patients in this study. ROIs, regions of interest; TNs, tumor nests; WSIs, whole‐slide images.

The main steps of feature extraction and prognostic model establishment based on PMP PIs proposed in this study are described in Figure 1.

### 2.6 Statistical analysis

X‐Tile was used to convert the continuous variables (including clinicopathological characteristics and image features extracted by QuPath) into categorical variables for subsequent survival analysis. Categorical variables were presented as numbers (percentages), and Spearman correlation analysis was used for correlation analysis. Then Kaplan–Meier survival analysis screened for features with potential prognostic significance, and then partial least squares (PLS) regression was performed for multiple correlation features dimensionality reduction. The optimal number of components in dimensionality reduction were obtained by the cross‐validation of root‐squared error of prediction (RMSEP) and variance explained rate for different number of components. The smaller the RMSEP, the larger the variance explained rate (close to one), indicating the better the number of components.^14^ Kaplan–Meier survival analysis was used to plot the survival curves between subgroups of features, and log‐rank test was used to calculate the statistical significance. Cox proportional regression model was applied to identify independent prognostic factors on OS. After multivariate survival analysis, a nomogram was constructed using independent predictors by “rms” R package to visually predict the 1‐, 2‐, and 3‐year survival probability of OS. The variable with the largest influence on the OS was assigned a maximum of 100 points, and other variables were assigned a lower maximum value proportional to their impacts. Concordance index (C‐index), calibration plots were used to evaluate the performance of the nomogram. C‐index was applied for assessment of discrimination ability of prediction model, and calibration plots was performed to determine the concordance between the predictive and actual survival at 1, 2, and 3 years. A two‐sided *p* < 0.05 was considered as statistically significant. All statistical analyses were performed with SPSS version 24.0 statistical software (IBM SPSS Inc., USA), R 4.3.0 software (https://www.r‐project.org/).

## 3 RESULTS

### 3.1 Major clinicopathological characteristics of PMP patients

Among the 92 PMP patients, there were 34 (37.0%) females and 58 (63.0%) males, with a median age of 57 (range: 31–76). Histologically, 45 (48.9%) patients were LMCP, 35 (38.0%) were HMCP, and 12 (13.0%) were HMCP‐S. Clinically, 49 (53.3%) patients had PCI ≤ 32, and 23 (25.0%) cases >36. The CC score was 0–1 in 44 (47.8%) cases and 2–3 in 48 (52.2%) cases. Kaplan–Meier survival analysis showed that BMI, Karnofsky performance status (KPS) score, preoperative CA199, histopathological type, vascular emboli, lymphatic metastasis, PCI score, and CC score were significantly associated with OS (*p* < 0.05) (Table 1).

**TABLE 1:** Major clinicopathological characteristics of PMP patients.

Variable	Value (%)	Median OS (month)	95% CI	*p* value
Gender				
Female	34 (37.0)	–	–	0.153
Male	58 (63.0)	44.7	25.7–63.7	
Age (years)				
≤49	21 (22.8)	34.5	25.3–43.8	0.074
>49 to ≤55	20 (21.7)	59.0	17.8–100.3	
>55	51 (55.4)	79.3	31.3–127.3	
BMI (kg/m^2^)				
≤18.5	8 (8.7)	21.6	0.0–43.3	**0.020**
>18.5 to ≤23.9	44 (47.8)	44.7	26.2–63.2	
>23.9	40 (43.5)	65.9	42.1–89.7	
KPS score				
≤80	28 (30.4)	29.8	21.1–38.5	**0.002**
>80 to ≤90	32 (34.8)	35.4	6.6–64.3	
>90	32 (34.8)	–	–	
Preoperative CEA				
Normal	9 (9.8)	–	–	0.180
Increased	83 (90.2)	53.0	33.1–72.9	
Preoperative CA199				
Normal	43 (46.7)	–	–	**0.001**
Increased	49 (53.3)	34.2	26.3–42.2	
Preoperative CA125				
Normal	25 (27.2)	53.5	–	0.144
Increased	67 (72.8)	46.8	22.5–71.1	
History of antitumor therapy				
No	66 (71.7)	59.0	39.0–79.1	0.182
Yes	26 (28.3)	35.4	19.9–51.0	
Histopathological type				
LMCP	45 (48.9)	79.3	–	**<0.001**
HMCP	35 (38.0)	46.8	20.7–72.9	
HMCP‐S	12 (13.0)	25.1	11.4–38.9	
Vascular emboli				
No	90 (97.8)	53.5	38.1–68.8	**0.021**
Yes	2 (2.2)	11.9	–	
Lymphatic metastasis				
No	83 (90.2)	59.0	43.3–74.8	**<0.001**
Yes	9 (9.8)	18.4	1.5–35.3	
PCI score				
≤32	49 (53.3)	65.9	–	**0.011**
>32 to ≤36	20 (21.7)	36.5	26.3–46.7	
>36	23 (25.0)	31.7	21.9–41.5	
CC score				
0–1	44 (47.8)	65.9	–	**0.001**
2–3	48 (52.2)	30.5	23.8–37.2	
SAEs				
No	61 (66.3)	44.7	25.1–64.3	0.724
Yes	31 (33.7)	60.7	27.5–94.0	

*Note*: Bold values indicates *p* < 0.05.

Abbreviations: BMI, body mass index; CA, carbohydrate antigen; CC, completeness of cytoreduction; CEA, carcinoma embryonic antigen; HMCP, high‐grade mucinous carcinoma peritonei; HMCP‐S, HMCP with signet ring cells; KPS, Karnofsky performance status; LMCP, low‐grade mucinous carcinoma peritonei; PCI, peritoneal cancer index; PMP, pseudomyxoma peritonei; SAEs, serious adverse events.

### 3.2 PIs feature acquisition

In this study, a total of 2245 patches were selected from 449 WSIs of 92 PMP patients. There were 1,208,717 cells detected, among which TCs, stromal cells, immune cells, and blood cells were 31.87%, 47.87%, 11.02% and 9.24%, respectively.

Forty image parameters of different components were extracted from multiple classes. These parameters were divided as tissue‐level features (including the number, size, and shape of TNs, etc., *n* = 11) and cell‐ and nucleus‐level features (including the density, size, shape, contour, etc., *n* = 29) based on feature acquisition approach (Table S1). The extracted quantized numerical features were converted into three categorical variables using X‐Tile.

### 3.3 Clinical value of morphologic parameters from PIs

The above extracted parameters were included in the log‐rank test, and the results showed as follows. At the tissue‐level, univariate survival analysis demonstrated statistically significant differences on OS between the subgroups of the following nine parameters: TNs number (*p* < 0.001), TNs area average (*p* < 0.001), TNs area variance (*p* = 0.001), TNs perimeter average (*p* < 0.001), TNs area sum (*p* = 0.029), TNs perimeter sum (*p* < 0.001), TNs area/perimeter (*p* < 0.001), TNs/stromal area ratio (*p* < 0.001), and TNs cell density (*p* < 0.001) (Table 2).

**TABLE 2:** Analysis of tissue‐level features regarding OS (*p* < 0.05).

Variable	Value (%)	Median OS (month)	95% CI	*p* value
TNs number				
≤14	42	–	–	<0.001
>14 to ≤41	36	41.6	25.4–57.8	
>41	14	19.0	2.4–35.6	
TNs area average				
≤2806.5	11	19.0	8.9–29.1	<0.001
>2806.5 to ≤6181.6	19	29.7	2.5–57.0	
>6181.6	62	78.6	57.1–100.0	
TNs area variance				
≤5111.3	15	23.0	9.5–36.6	0.001
>5111.3 to ≤8289.6	21	41.6	14.5–68.7	
>8289.6	56	78.6	58.1–99.1	
TNs perimeter average				
≤372.8	13	19.0	8.0–30.0	<0.001
>372.8 to ≤582.3	19	53.0	19.2–86.8	
>582.3	60	78.6	52.5–104.6	
TNs area sum				
≤150,426.9	60	59.0	35.9–82.1	0.029
>150,426.9 to ≤197,517.7	21	36.5	20.6–52.4	
>197,517.7	11	25.1	4.7–45.5	
TNs perimeter sum				
≤13,503.1	61	65.9	42.7–89.2	<0.001
>13,503.1 to ≤17,729.0	19	34.2	27.1–41.4	
>17,729.0	12	12.6	0.0–29.1	
TNs area/perimeter ratio				
≤7.5	21	21.9	13.3–30.4	<0.001
>7.5 to ≤8.0	20	53.5	34.2–72.8	
>8.0	51	–	–	
TNs/stromal area ratio				
≤0.01	8	19.0	6.5–31.5	<0.001
>0.01 to ≤0.03	27	30.4	3.8–57.1	
>0.03	57	78.6	58.0–99.2	
TNs cell density				
≤987	49	78.6	–	<0.001
>987 to ≤1340	25	59.0	24.0–94.0	
>1340	18	21.6	14.4–28.8	

Abbreviations: OS, overall survival; TNs, tumor nests.

At the cell‐level, there were statistically significant differences on OS among the subgroups of TCs area variance (*p* = 0.007), TCs perimeter variance (*p* = 0.036), TCs area/perimeter ratio (*p* = 0.029), and pericancerous blood cell density (*p* = 0.015) (Table 3). At the nucleus‐level, there were statistically significant differences on OS among the subgroups of TCs nuclei area average (*p* = 0.001), TCs nuclei area variance (*p* = 0.003), TCs nuclei perimeter average (*p* = 0.015), TCs nuclei circularity variance (*p* = 0.027), the min caliper average (*p* = 0.005), variance (*p* = 0.001) of TCs nuclei, and TCs nuclei area/perimeter ratio (*p* = 0.015) (Table 4).

**TABLE 3:** Analysis of cell‐level features regarding OS (*p* < 0.05).

Variable	Value (%)	Median OS (month)	95% CI	*p* value
TCs area variance				
≤54.10	56	59.0	44.4–73.7	0.007
>54.10 to ≤59.68	24	46.8	19.0–74.6	
>59.68	12	28.5	19.3–37.6	
TCs perimeter variance				
≤10.62	61	59.0	43.8–74.2	0.036
>10.62 to ≤11.27	19	46.8	19.7–73.9	
>11.27	12	28.5	19.3–37.6	
TCs area/perimeter ratio				
≤2.32	16	–	–	0.029
>2.32 to ≤2.47	48	59.0	37.7–80.3	
>2.47	28	30.5	12.4–48.6	
Pericancerous blood cell density				
≤84	25	–	–	0.015
>84 to ≤155	21	41.6	21.4–61.8	
>155	46	36.5	22.8–50.2	

Abbreviations: OS, overall survival; TCs, tumor cells.

**TABLE 4:** Analysis of nucleus‐level features regarding OS (*p* < 0.05).

Variable	Value (%)	Median OS (month)	95% CI	*p* value
TCs nuclei area average				
≤29.72	49	59.0	32.9–85.1	0.001
>29.72 to ≤30.84	19	78.6	44.8–112.3	
>30.84	24	28.5	21.4–35.6	
TCs nuclei area variance				
≤19.67	40	53.5	36.2–70.8	0.003
>19.67 to ≤23.40	35	78.6	–	
>23.40	17	28.7	21.8–37.6	
TCs nuclei perimeter average				
≤23.55	57	60.7	45.4–76.1	0.015
>23.55 to ≤24.33	19	–	–	
>24.33	16	29.7	17.6–41.8	
TCs nuclei circularity variance				
≤0.139	15	21.9	6.7–37.0	0.027
>0.139 to ≤0.143	42	46.8	22.4–71.2	
>0.143	35	78.6	43.1–114.1	
The minor axis average of TCs nuclei ellipse				
≤4.95	54	65.9	40.7–91.2	0.005
>4.95 to ≤5.17	24	53.5	27.8–79.1	
>5.17	14	24.3	9.9–38.7	
The minor axis variance of TCs nuclei ellipse				
≤1.74	47	59.0	43.3–74.7	0.001
>1.74 to ≤1.91	30	–	–	
>1.91	15	28.5	21.7–35.3	
TCs nuclei area/perimeter ratio				
≤1.17	29	65.9	37.6–94.3	0.015
>1.17 to ≤1.22	46	53.0	29.5–76.6	
>1.22	17	29.7	15.9–43.6	

Abbreviations: OS, overall survival; TCs, tumor cells.

### 3.4 Screening for PIs features

#### 3.4.1 Correlation analysis

**FIGURE 2:**
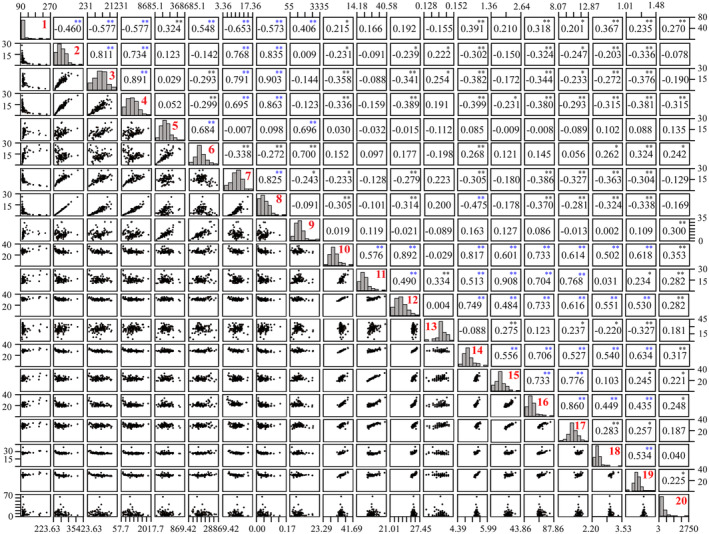
Pair wise correlation and scatter plot matrix of all significant variables in KM analysis. The correlation between variables (super‐diagonal), two‐way scatter plot (sub‐diagonal), and the histogram of each variable (diagonal). Red number: 1: TNs number; 2: TNs area average; 3: TNs area variance; 4: TNs perimeter average; 5: TNs area sum; 6: TNs perimeter sum; 7: TNs area/perimeter ratio; 8: TNs/stromal area ratio; 9: TNs cell density; 10: TCs nuclei area average; 11: TCs nuclei area variance; 12: TCs nuclei perimeter average; 13: TCs nuclei circularity variance; 14: the min caliper average of TCs nuclei; 15: the min caliper variance of TCs nuclei; 16: TCs area variance; 17: TCs perimeter variance; 18: TCs area/perimeter ratio; 19: TCs nuclei area/perimeter ratio; 20: pericancerous blood cell density. Blue *: there was a stronger correlation between the two variables (|*r*|>0.5, *p* < 0.001).

Since features extracted in the process of machine learning were prone to product relevant features, the Spearman correlation analysis was carried out on the above 20 parameters (including nine at tissue‐level, four at cell‐level, and seven at nucleus‐level). Figure 2 simultaneously showed the correlation between parameters (super diagonal), two‐way scatter plot (sub diagonal), and the histogram of each parameter (diagonal). The correlation coefficient >0.5 was determined as close relation between two parameters. The results showed that there were multiple significant correlations among the nine parameters at tissue‐level; there were multiple significant correlations among the nine parameters at cell‐ and nucleus‐level except TCs nuclei circularity variance and pericancerous blood cell density (|*r*|>0.5, *p* < 0.001).

#### 3.4.2 PLS regression

**FIGURE 3:**
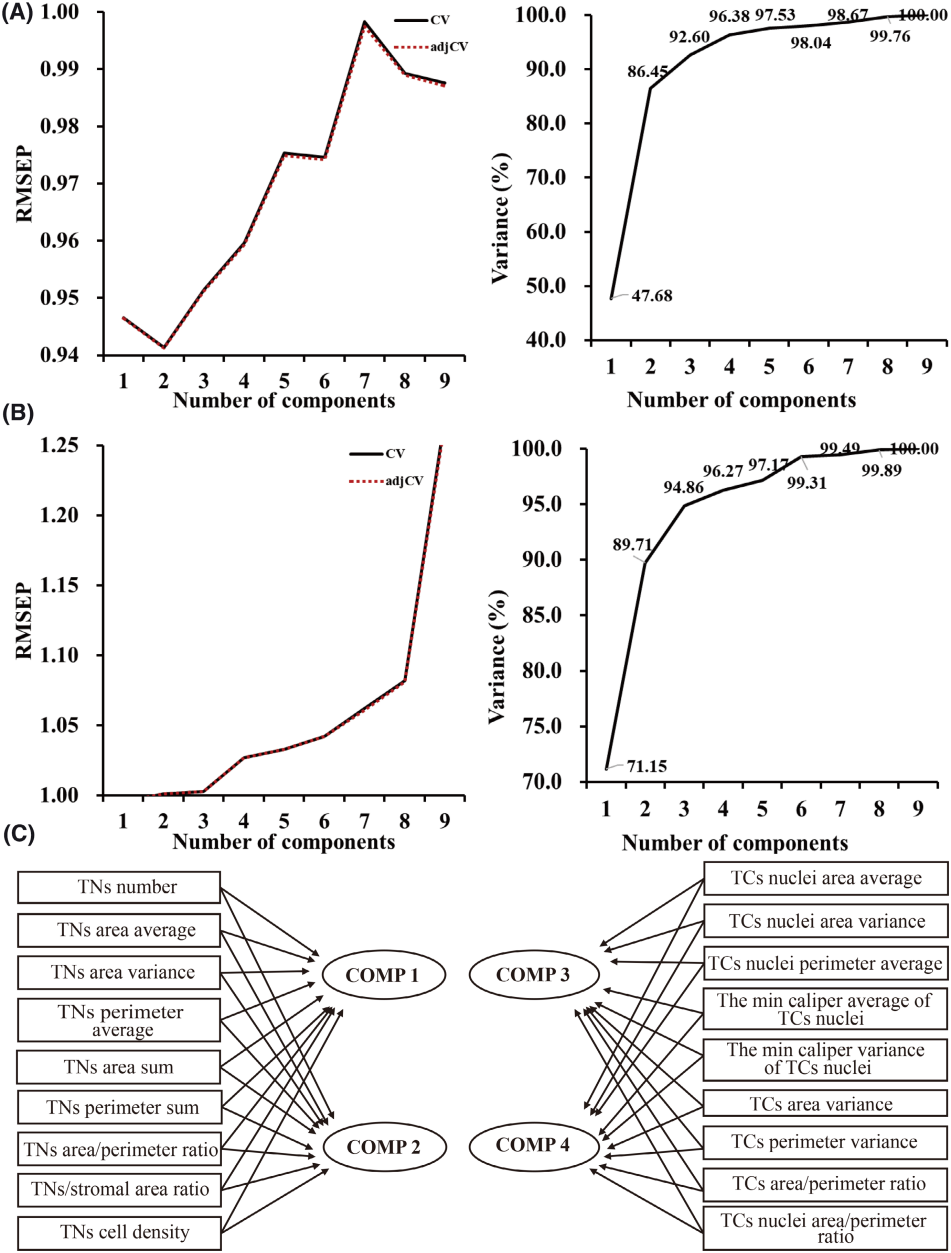
PLS regression. (A) RMSEP and variance explained rate for different number of components of tissue‐level features; (B) RMSEP and variance explained rate for different number of components of cell‐ and nucleus‐level features; (C) PLS workflow. COMP, component; PLS, partial least squares; RMSEP, root‐squared error of prediction; TCs, tumor cells; TNs, tumor nests.

Multiple correlation parameters of the above two levels were included into PLS regression respectively for feature dimensionality reduction. According to the results of RMSEP and variance explained rate, it was found that the PLS regression model at tissue‐level had a better performance when it contains two components (RMSEP is relatively small and variance explained rate exceeded 85.0%). Similarly, the regression model at cell‐ and nucleus‐level worked better when it had two components (Figure 3A,B). As a result, four new parameters were obtained by PLS regression: component (COMP) 1 and COMP 2 at tissue‐level, COMP 3, and COMP 4 at cell‐ and nucleus‐level (Figure 3C). Table S2 presented the PLS regression coefficient with four components.

### 3.5 Clinical value of image features by multivariate analysis

#### 3.5.1 Overall survival curve analysis

**FIGURE 4:**
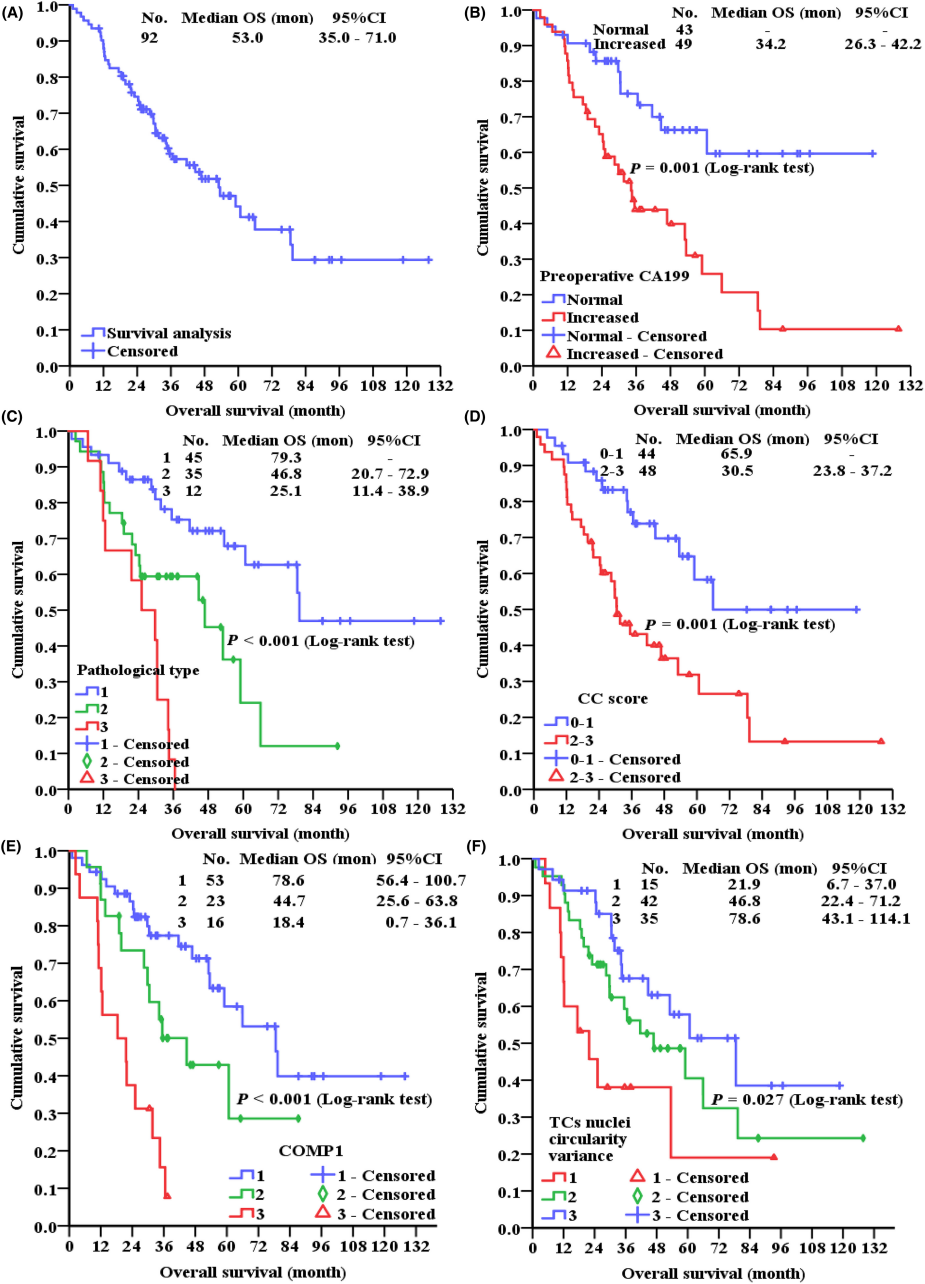
Kaplan–Meier curves of all patients. (A) Survival analysis; (B) preoperative CA199: (C) histopathological type (1: LMCP; 2: HMCP; 3: HMCP‐S); (D) CC score; (E) COMP 1 (1: ≤18,622.19; 2:18,622.19–26,351.96; 3: >26,351.95); (F) TCs nuclei circularity variance (1: ≤0.139; 2:0.139–0.143; 3: >0.143). OS, overall survival; LMCP, low‐grade mucinous carcinoma peritonei; HMCP, high‐grade mucinous carcinoma peritonei; HMCP‐S, HMCP with signet ring cells; CC, completeness of cytoreduction; COMP, component; TCs, tumor cells.

The median follow‐up time was 52.3 months (95% CI: 43.3–61.3), and the median OS was 53.0 months (95% CI: 35.0–71.0 months). Forty‐six patients (50.0%) died and forty‐six (50.0%) survived. The 1‐, 2‐, 3‐, and 5‐year survival rates were 88.0%, 74.6%, 58.8%, and 44.2%, respectively (Figure 4A).

#### 3.5.2 Multivariate survival analysis

To verify the clinical value of the newly selected image features, these parameters were combined with traditional histopathological characteristics for multivariate survival analysis. Similarly, in order to avoid possible multicollinearity between variables in the COX regression model, Spearman rank correlation analysis was performed, and the results showed that there was no close correlation between image parameters and general pathological characteristics such as histopathological type (|*r*| < 0.5).

Then, factors in the univariate survival analysis (*p* < 0.05) were incorporated into the COX regression model, delineating the following five independent prognostic indicators. In addition to three traditional factors including preoperative CA199, CC score and histopathological type, two image features including COMP 1 at tissue‐level and TCs nuclei circularity variance (Figure 4; Table 5).

**TABLE 5:** Multivariate COX proportional hazards model in 92 PMP patients.

Variable	Hazard ratio	95% CI	*p* value
Preoperative CA19‐9	2.033	1.034–4.000	0.040
CC score	3.803	1.903–7.598	<0.001
Histopathological type	–	–	0.001
(HMCP vs. LMCP)	2.578	1.202–5.527	0.015
(HMCP‐S vs. LMCP)	6.401	2.338–17.522	<0.001
COMP 1 in tissue‐level			0.053
(18,622.19–26,351.96 vs. ≤18,622.19)	2.200	1.014–4.774	0.046
(>26,351.95 vs. ≤18,622.19)	2.721	1.086–6.817	0.033
TCs nuclei circularity variance			0.013
(0.139–0.143 vs. ≤0.139)	0.399	0.173–0.921	0.031
( >0.143 vs. ≤0.139)	0.238	0.092–0.621	0.003

Abbreviations: BMI, body mass index; CC, completeness of cytoreduction; COMP, component; HMCP, high‐grade mucinous carcinoma peritonei; HMCP‐S, HMCP with signet ring cells; KPS, Karnofsky performance status; LMCP, low‐grade mucinous carcinoma peritonei; PCI, peritoneal cancer index; PMP, pseudomyxoma peritonei; TCs, tumor cells.

#### 3.5.3 Establishment and evaluation of nomogram

**FIGURE 5:**
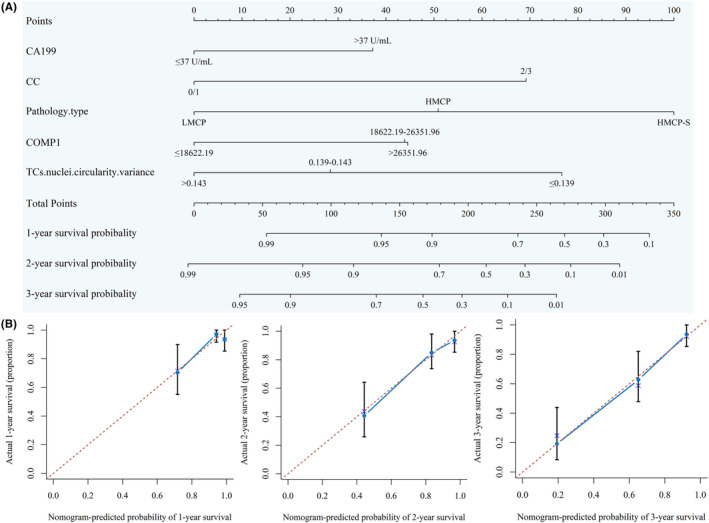
Establishment of nomogram of PMP patients. (A) Construction of prognostic nomogram to predict survival of patients; (B) calibration plots of the nomogram for predicting 1‐, 2‐, and 3‐year survival probability. CA199, carcinoembryonic antigen 199; CC, completeness of cytoreduction; COMP, component; PMP, pseudomyxoma peritonei.

To construct a clinical‐based method for predicting the prognosis of PMP patients, a nomogram that incorporated the above five independent prognostic factors was established, and the C‐index was 0.795 (95% CI: 0.748–0.842). The Calibration plots (1000 bootstrap resamples) showed highly consistent between the predicted and actual observation in predicting 1‐year, 2‐year, and 3‐year survival rate, indicating an improved predictive performance of nomogram (Figure 5).

## 4 DISCUSSION

In this study, a total of 449 HE‐stained WSIs of 92 PMP patients were marked and characterized quantitatively using QuPath. Forty detailed morphological parameters were extracted from the tissue‐, cell‐ and nuclei‐level. Kaplan–Meier analysis showed that 20 features were significantly correlated with OS, and they were fused into four composite features by correlation analysis and PLS regression. Sub visual pathological features and other clinicopathological indicators related to prognosis were incorporated into COX regression, and it identified five independent prognosticators of three categories: (1) preoperative tumor marker: CA199; (2) operation technique: CC score; (3) tumor biological characteristics: Histopathological type, COMP 1 at tissue‐level, and TCs nuclei circularity variance. The nomogram demonstrated that the effect on prognosis in descending order was as follows: Histopathological type, TCs nuclei circularity variance, CC score, COMP 1 and preoperative CA199. The validation results showed that the C index reached 0.795, and the calibration curve also showed good prediction performance in 1‐, 2‐, and 3‐year survival rate.

PIs researches of other tumors have shown that HE stained PIs contained many potential prognostic features.^15^, ^16^ In contrast, the in‐depth studies of PMP pathology are relatively rare, and only some investigators have tried quantitatively analysis on PIs. Some studies have shown that TCs density and morphology could affect the prognosis of PMP,^17^, ^18^, ^19^ and stromal components also played an important role of PMP prognosis.^8^, ^20^ However, these studies were qualitative or semi‐quantitative with relatively simple evaluations, which failed to comprehensive analyze the whole PIs. Moreover, most studies focused on the tumor, and stromal research was not enough. In addition, reliable prediction tools for PMP, such as nomogram, are relatively scarce. Several previous studies using nomogram have shown that C‐index ranges from 0.740 to 0.825.^21^, ^22^, ^23^ The nomogram in this study also showed good prediction performance (C‐index was 0.795). Previous studies included the following features: In multi‐center SEER dataset study, some prognostic factors were missed in dataset and excluded in nomogram^23^; In single‐center large‐sample study, some important data were missing due to the long‐time span or patient's referral^21^; or other small‐sample studies with complete data.^22^ Moreover, these prognostic models only included general clinicopathological features. It is important to explore potential features from abundant PIs information and establish a nomogram prediction model to assist clinical decisions for PMP patients.

In this study, we found five independent prognosticators, and a nomogram was developed for prognosis prediction and assess the risk for PMP patients. Preoperative CA199 was an independent prognostic factor, like previous studies. The biomarker CA199 can inhibit cell differentiation, promote cytoadherence, and enhance tumor metastasis, thus judging the proliferation activity of TCs.^24^ And some previous studies have also demonstrated that high preoperative CA199 level could serve as an independent factor for poor prognosis.^25^, ^26^ Moreover, Hiraide et al.^27^ have found that modified FOLFOX6 chemotherapy could decrease CA199 level, so reducing preoperative CA199 through perioperative chemotherapy may provide a way to improve the prognosis of PMP patients.

Another independent prognosticator was CC score, which similar to our previous study.^28^ CC score is an objective index to evaluate tumor resection of standardized CRS, and previous studies found PMP patient with satisfied CC score had better prognosis.^22^, ^29^ Huang et al.^30^ assessed the learning curve of CRS + HIPEC technique and found that CC0 complete rate increased with accumulation of surgical experience. Bai et al.^31^ found that gender, disease duration, anemia and preoperative CA199 could help to predict CC score in PMP patient. Passot et al.^32^ found that preoperative 18F‐FDG PET could predict the postoperative CC score of PMP patient. Therefore, standardized CRS + HIPEC; surgical techniques improvement, and the professional examination before operation could decrease the postoperative CC score and thus improve the survival.

We also found that histopathological type was an independent prognosticator, and had the greatest impact on PMP prognosis based on nomogram. The histopathological classification is essential for the assessment of tumor behavior. Previous researchers also found that histopathological type could significantly affect the prognosis of PMP patients.^33^ In contrast, some studies have found no significant correlation between the prognosis with histopathological type,^34^ and studies have even showed some PMP patients with poor malignant grade had a better prognosis than those with better differentiation.^7^ The difference may come from the standardization of PMP classification criteria, the difference of observers. Moreover, as mentioned above, PMP PIs may contain abundant information to explore new pathological prognostic features.

In this study, QuPath was used to analyze PMP PIs. Multiple morphological features were extracted at tissue‐level to measure the morphological complexity of TNs structure; And the size, shape, contour of TCs and nuclei were extracted at cell‐ and nucleus‐level to quantify the polymorphism of TCs. This could help to overcome the inconsistency of subjective interpretation, quantitatively extract features as objectively and repeatably as possible; Moreover, with digital PIs features as the core, the prognosis model was established and two new prognostic features beyond conventional pathological parameters were found.

Tumor invasion largely depends on the collective behavior of TCs populations, that is, TNs. In‐depth study of TNs and TCs can reveal more useful information about tumor development. Our previous study showed that the ratio of tumor/stroma area had a significant impact on the prognosis, suggesting the clinical value of TNs area.^35^ Choudry et al.^19^ and Horvath et al.^36^ found that PMP patients with medium or high TCs density had a higher risk of disease progression. Bhatt et al.^18^ found that the cytological morphology of TCs also affected the prognosis of PMP patients. Our study explored more features based on previous studies. The dimension‐reduction features COMP 1 and COMP 2 of PIs comprehensively evaluated TNs morphology. Survival analysis showed that COMP 1 was an independent prognosticator, again verifying that TNs behavior was closely related to the malignancy of PMP.

In addition, the nomogram showed TCs nuclei circularity variance had a greater impact on OS than COMP 1. The changes of TCs nucleus plays a leading role in tumor occurrence, development and metastasis, and the morphological changes in PIs are an easy and intuitive method to detect the transforms of tumor nucleus.^37^ Nuclear characteristics in PIs are basis for benign or malignant detection and grading of many solid tumors. Papanicolaou's smear test is used to diagnose uterine and cervical cancer by detecting nuclear chromatin staining, size, and shape changes.^38^ Whitney et al.^39^ found that nuclear shape, texture and structure could independently predict the risk of recurrence in ER+ breast cancer patients. This study showed that TCs nuclei circularity variance, which mainly evaluated the uniformity of nuclear roundness, was an independent prognosticator, and the greater the difference in nuclear roundness, the better the prognosis of PMP patients.

Although some promising results have been obtained in this study, we must acknowledge the innate limitations of this exploratory work. First, the relatively small sample size inevitably reduces the representing power of the cohort, which could be a source of potential bias. Second, our nomogram was only verified by internal validation, which is inevitably less convincing than external validation. Therefore, it is necessary to conduct further prospective model verification including a larger sample from multi‐center database. Third, the molecular map of genomic data is also important for tumor prognosis, and it is better if multiple omics data can be integrated to establish a prognostic model. Finally, the quantitative research of HE stained PIs of PMP is still in exploratory stage, and the number and quality of sub visual features extracted may be limited. In the future, better algorithms need to be developed to excavate more potential pathological features.

In conclusion, this study established a prognosis nomogram prediction model based on new pathological characteristics of PMP through quantitative analysis of PIs, which represents the first one step forward towards digitalized pathological diagnosis in PMP routine pathology. This work could enhance the efficiency and accuracy in routine clinicopathologic diagnosis, significantly reduce the manual labor and more objectively locate the typical pathological ROIs and evaluate the importance impact of pathological features on the PMP prognosis, which is conductive to more accurate pathological diagnosis, disease course prediction and treatment decision. It is supposed that the key to further improve the prognosis of PMP in the future may be: (1) new adjuvant therapy to reduce preoperative CA 199; (2) more thorough CRS; (3) improving the ability to explore the information of the occurrence and development of PMP tumor itself to predict the future biological behavior is an important and urgent clinical task.

## AUTHOR CONTRIBUTIONS

**Ru Ma:** Conceptualization (equal); data curation (lead); formal analysis (lead); investigation (lead); methodology (lead); project administration (equal); software (equal); supervision (equal); validation (equal); visualization (equal); writing – original draft (lead); writing – review and editing (lead). **Yan‐Dong Su:** Data curation (equal); methodology (equal); project administration (equal); software (equal); supervision (supporting); visualization (equal); writing – review and editing (equal). **Feng‐Cai Yan:** Data curation (supporting); investigation (equal); resources (lead); supervision (supporting); writing – review and editing (equal). **Yu‐Lin Lin:** Data curation (supporting); methodology (supporting); software (equal); supervision (supporting); writing – review and editing (equal). **Ying Gao:** Data curation (supporting); project administration (supporting); resources (lead); supervision (supporting); writing – review and editing (equal). **Yan Li:** Conceptualization (lead); formal analysis (equal); funding acquisition (lead); investigation (equal); project administration (lead); resources (equal); supervision (lead); validation (equal); writing – review and editing (lead).

## FUNDING INFORMATION

This manuscript was supported by the General Program of National Natural Science Foundation of China, no. 82073376.

## CONFLICT OF INTEREST STATEMENT

The authors declare that they have no competing interests.

## ETHICS STATEMENT

This study was performed in line with the principles of the Declaration of Helsinki. Approval was granted by the Ethics Committee of Beijing Shijitan Hospital, Capital Medical University [2015 Research Ethics Review No. (28)].

## PATIENT CONSENT STATEMENT

Informed consent was obtained from all individual participants included in the study.

## Supporting information


Table S1.

Table S2.

## Data Availability

The datasets used and/or analyzed during the current study are available from the corresponding author on reasonable request.
