# Supplementary material for: A nomogram prediction model of pseudomyxoma peritonei established based on new prognostic factors of HE stained pathological images analysis
Source: Cancer Med. 2024 Mar 20;13(6):e7101. doi: 10.1002/cam4.7101 (PMC10952024; doi:10.1002/cam4.7101)
Supplement: Supplementary file 1 — Table S1. [file CAM4-13-e7101-s001.docx]

| **Table S1**. The features extracted from 92 PMP patients | | |
| --- | --- | --- |
| Level | Feature | Number |
| Tissue-level | TNs number | 11 |
|  | TNs area average |  |
|  | TNs area variance |  |
|  | TNs perimeter average |  |
|  | Centroid X |  |
|  | Centroid Y |  |
|  | TNs area sum |  |
|  | TNs perimeter sum |  |
|  | TNs area/perimeter |  |
|  | TNs/stromal area |  |
|  | TNs cell density |  |
| Cell-level | Area average | 10 |
|  | Area variance |  |
|  | Perimeter average |  |
|  | Perimeter variance |  |
|  | Area/perimeter |  |
|  | Immune cells density |  |
|  | Blood cells density |  |
|  | Other stroma cells density |  |
|  | Immune/tumor cells density ratio |  |
|  | Blood/tumor cells density ratio |  |
| Nucleus-level | Area average | 19 |
|  | Area variance |  |
|  | Perimeter average |  |
|  | Perimeter variance |  |
|  | Circularity average |  |
|  | Circularity variance |  |
|  | Min caliper average |  |
|  | Min caliper variance |  |
|  | Max caliper average |  |
|  | Max caliper variance |  |
|  | Eccentricity average |  |
|  | Eccentricity variance |  |
|  | Area/perimeter ratio |  |
|  | Nucleus/cell area ratio |  |
|  | Nuclear-cytoplasmic ratio |  |
|  | Stroma nucleus area average |  |
|  | Stroma/immune nucleus area ratio |  |
|  | Stroma nucleus circularity |  |
|  | Immune nucleus circularity |  |
| PMP: Pseudomyxoma peritonei; TNs: Tumor nests; TCs: Tumor cells | | |
