# Supplementary material for: A nomogram prediction model of pseudomyxoma peritonei established based on new prognostic factors of HE stained pathological images analysis
Source: Cancer Med. 2024 Mar 20;13(6):e7101. doi: 10.1002/cam4.7101 (PMC10952024; doi:10.1002/cam4.7101)
Supplement: Supplementary file 2 — Table S2. [file CAM4-13-e7101-s002.docx]

| **Table S2**. The results of screening the multicollinearity features by PLS regression | | | | |
| --- | --- | --- | --- | --- |
| Variable | Tissue-level | | Cell- and nucleus-level | |
|  | COMP 1 *^a^* | COMP 2 *^b^* | COMP 3 *^c^* | COMP 4 *^d^* |
| TNs number | 0.341 | - | - | - |
| TNs area average | -0.382 | 0.159 | - | - |
| TNs area variance | -0.225 | 0.446 | - | - |
| TNs perimeter average | -0.397 | - | - | - |
| TNs area sum | 0.149 | 0.593 | - | - |
| TNs perimeter sum | 0.341 | 0.300 | - | - |
| TNs area/perimeter ratio | -0.407 | 0.156 | - | - |
| TNs/stromal area ratio | -0.366 | 0.177 | - | - |
| TNs cell density | 0.303 | 0.516 | - | - |
| TCs nuclei area average | - | - | 0.416 | 0.167 |
| TCs nuclei area variance | - | - | 0.293 | -0.214 |
| TCs nuclei perimeter average | - | - | 0.348 | -0.194 |
| The min caliper average of TCs nuclei | - | - | 0.484 | 0.307 |
| The min caliper variance of TCs nuclei | - | - | 0.259 | -0.310 |
| TCs area variance | - | - | 0.360 | -0.262 |
| TCs perimeter variance | - | - | 0.124 | -0.657 |
| TCs area/perimeter ratio | - | - | 0.226 | - |
| TCs nuclei area/perimeter ratio | - | - | 0.350 | 0.441 |
| PLS: Partial least-squares; COMP: Component; TNs: Tumor nests; TCs: Tumor cells; *^a^* COMP 1 in tissue-level=0.341×TNs number-0.382×TNs area average-0.225×TNs area variance-0.397×TNs perimeter average+0.149×TNs area sum+0.341×TNs perimeter sum-0.407×TNs area/perimeter ratio-0.366×TNs/stromal area ratio+0.303×TNs cell density; *^b^* COMP 2 in tissue-level=0.159×TNs area average+0.446×TNs area variance+0.593×TNs area sum+0.300×TNs perimeter sum+0.156×TNs area/perimeter ratio+0.177×TNs/stromal area ratio+0.516×TNs cell density; *^c^* COMP 3 in cell- and nucleus-level=0.416×TCs nuclei area average+0.293×TCs nuclei area variance+0.348×TCs nuclei perimeter average+0.484×The minor axis average of TCs nuclei ellipse+0.259×The minor axis variance of TCs nuclei ellipse+0.360×TCs area variance+0.124×TCs perimeter variance+0.226×TCs area/perimeter ratio+0.350×TCs nuclei area/perimeter ratio; *^d^* COMP 4 in cell- and nucleus-level=0.167×TCs nuclei area average-0.214×TCs nuclei area variance-0.194×TCs nuclei perimeter average+0.307×The minor axis average of TCs nuclei ellipse-0.310×The minor axis variance of TCs nuclei ellipse-0.262×TCs area variance-0.657×TCs perimeter variance+0.441×TCs nuclei area/perimeter ratio | | | | |
